# Editorial: Glia-Mediated Neurotoxicity: Uncovering the Molecular Mechanisms

**DOI:** 10.3389/fnmol.2022.980763

**Published:** 2022-07-18

**Authors:** Amit K. Srivastava, Barbara Lukomska, Lorraine Iacovitti

**Affiliations:** ^1^Department of Medicine, Sidney Kimmel Medical College, Thomas Jefferson University, Cardeza Foundation for Hematologic Research, Philadelphia, PA, United States; ^2^NeuroRepair Department, Mossakowski Medical Research Institute, Polish Academy of Sciences, Warsaw, Poland; ^3^Department of Neuroscience, Vickie and Jack Farber Institute for Neuroscience, Jefferson Stem Cell and Regenerative Neuroscience Center, Thomas Jefferson University, Philadelphia, PA, United States

**Keywords:** glia, neurotoxicity, neurodegeneration, central nervous system (CNS), molecular mechanism

Glial cells, including non-neuronal cells of the neuroepithelium (oligodendrocytes, astrocytes, oligodendrocyte progenitor cells, ependymal cells), neural crest (peripheral glia), and of myeloid origin (microglia), constitute a large fraction of the central nervous system (CNS). Glia was initially described in 1856 by pathologist Rudolf Virchow as a “connective tissue” that binds nervous elements together (Parpura et al., 2012). During the course of the twentieth century, microglia were discovered and neuroscientists' views evolved toward considering glia only as auxiliary cells of neurons (von Bernhardi et al., 2016). For decades, glial cells were considered silent partners of CNS neuronal physiology. Now, it is well accepted that they are crucial players of the nervous system homeostasis, playing active regulatory role in immune surveillance, buffer the action of neurotransmitters, and secrete nerve growth factors and cytokines (Hamilton et al., 2007; Huizinga et al., 2012). In addition, glial cells are able to induce neurogenesis, regulate neuronal activity and synaptic transmission (Grubisic and Gulbransen, 2017). Glia-mediated neurotoxicity due to glia cells dysfunction has been known to play an important role in a wide range of neurological disorders however, its cellular and molecular mechanisms have yet to be fully understood. Gaining a greater understanding of glia-mediated neurotoxicity will not only provide insight into this critical biological event, but also be instrumental in advancing knowledge of brain development and neurological diseases. The current issue provides a platform for a critical review on recent advances and original articles that offer significant insights into biochemical and molecular aspects of glia-mediated neurotoxicity with the potential of identifying novel therapeutic targets.

Over the past several decades, a large amount of data revealed that the crosstalk between glial cells and neurons represents an exceptional feature for maintaining the normal function of CNS. Recently, extracellular vesicles (EVs) released by both glia and neurons were considered to play important roles in exchanging molecular information between neural cells. Extracellular vesicles have been shown to contain and transfer proteins and nucleic acids to target cells, modulating their functional activity (Koniusz et al., 2016). A review article by Ahmad et al. shed a new light on the role of extracellular (EVs) in glia and neuronal cells' communication and their roles in CNS physiology and pathology. The authors demonstrated that different types of glia and neuronal cells secrete various types of EVs, resulting in specific functions in intercellular communications.

Among glial cells, astrocytes are the most numerous cells in the central nervous system (Jakel and Dimou, 2017). They play a diverse role, regulating neuronal functions, oligodendroglia differentiation, blood-barrier maintenance, and homeostasis in the CNS. Astrocytes are engaged in various physiological processes including brain energy metabolism of glucose (Duran-Aniotz and Hetz, 2016). In glucose metabolism, part of glucose used to convert UDP-GlcNAc as a donor molecule for O-GlcNAcylation is controlled by O-GlcNAc transferase enzyme (OGT). The role of O-GlcNAcylation in astrocytes is almost completely unknown. Performing proteomic analysis of medial prefrontal cortex (mPFC) tissues obtained from astrocyte specific conditional OGT knockout mice, the studies presented in this issue by Fan et al. have shown that astrocytic OGT could alter the expression of proteins participating in metabolic processes, transferase activity, and biosynthetic processes. These results paved the path for further investigation of astrocytic OGT's role in the mPFC, which plays a critical role in numerous behavioral and cognitive functions.

In certain conditions, astrocytes respond to the injury, infection and inflammation by acquiring reactive astrogliosis, a pathological hallmark of CNS structural lesions (Sofroniew and Vinters, 2010). It is reported that microglia activated by neuro-inflammatory insults induce the conversion of resting astrocytes to reactive astrocytes secreting TNFα, IL-1α, and C1Cq11q (TIC). Thus, reactive astrocytes have been implicated as a source of various neurotoxic factors to neurons and oligodendrocytes (Di Giorgio et al., 2007; Liddelow et al., 2017). In this issue, Labib et al. presented a human induced pluripotent stem cell (hiPSC) model to investigate the surface marker profile and proteome of TIC-induced reactive astrocytes. The authors identified VCAM1M, BST2T, ICOSL, HLA-E, PD-L1L, and PDPN as novel markers of TIC-induced reactive astrocytes. Moreover, proteomic analysis of TIC-induced reactive astrocyte identified proteins and related pathways linked to engagement with peripheral immune cells (Labib et al.). These findings have potential to serve as new tools to identify reactive astrocyte subtypes in neurodegenerative diseases and investigate their involvement in glia-mediated neurotoxicity. Interestingly, the authors found that several of these markers co-localize with GFAP^+^ cells in post-mortem samples from people with Alzheimer's disease.

In another paper presented in this issue, Smith et al. explored the impact of reactive astrocytes on oligodendrocyte differentiation in a human system using human hiPSC-derived astrocytes and a human oligodendrocyte reporter cell line. The hypothesis underlying these experiments was to discern whether inherent differences exist in astrocytes related to multiple sclerosis (MS). They reported that the pro-inflammatory hiPSC-astrocyte secretome inhibited hESC-derived oligodendrocyte precursor cell (OPC) differentiation. Bulk transcriptome analysis of human OPC-enriched cultures identified significantly differentially regulated genes and pathways that may contribute to failed re-myelination phenotype (Smith et al.). This study adds important new information in terms of understanding generalizable astrocyte responses in MS.

In brief, research topic introduced in the special issue of Frontiers in Molecular Neuroscience highlights the role of glia-mediated toxicity in neurodegenerative disorders. As our understanding of glial cell biology grows, so too will the probability of developing effective therapeutic strategies.

## Author contributions

AS, BL, and LI wrote and edited the manuscript. All authors contributed to the article and approved the submitted version.

## Conflict of interest

The authors declare that the research was conducted in the absence of any commercial or financial relationships that could be construed as a potential conflict of interest.

## Publisher's note

All claims expressed in this article are solely those of the authors and do not necessarily represent those of their affiliated organizations, or those of the publisher, the editors and the reviewers. Any product that may be evaluated in this article, or claim that may be made by its manufacturer, is not guaranteed or endorsed by the publisher.
